# The Abuse of Plastic Fillings

**Published:** 1872-06

**Authors:** W. George Beers

**Affiliations:** Montreal.


### ARTICLE VIII.

### *The Abuse of Plastic Fillings.*

Read before the Montreal Dental Society, by W. George Beers, L.D.S, Montreal.

The ignorance or depravity of some men of the dental profession brings discredit upon its reputation—for there is no use flattering ourselves that our profession is exempt from members who are ignorant because uneducated, and quacks because constitutionally depraved. There is no operation in dentistry, but may be shamefully abused; and allowing for the stage of immaturity through which we all have to pass, and during which time we doubtless did work that would now bring a blush to the cheek, there is a frequent abuse of plastic fillings now extant to which I venture to draw attention for a few moments. It is dangerous to extol even a good principle or practice too much, because there is no principle or practice in dentistry but

3

has its limits and restrictions, and there are men in the profession who will carry a practice to any extreme, so long as it is convenient and cheap.

Many of the best operators and thinkers in our profession to-day are those who count their practice by decades, and whose intellectual and physical vigor overmatch the most of their compeers. But there are a few practitioners of the old school left who prefer an old way of doing a thing, lest the trial of a new way should involve more study and investigation than they care to give; who made up their minds to make the supply of dental knowledge obtained in their youth do them for the rest of their lives, and who have a superstitious respect for the dry bones and dead ideas of ancestors. They magnify men of their opinion, however obscure, into men of eminence, and dwindle those who differ with them, however famed, into nonentities. If they could have their way, experiment would never break the delusion of ignorance, and old opinions would be more weighty than modern investigation.

It is now generally conceded that those of the old opponents of amalgam who are progressive, not prejudiced, and who have the independence to think, test and observe for themselves, that the abuse of this plastic filling at the time of its introduction, by the use of an impure, uncleansed mixture of filed coin silver, which when amalgamated was seldom washed, and when used was never used well, led many to abstain from or abandon its use, and that thousands of good teeth were then extracted by leading practitioners which no other filling would save. It is also conceded that many cases exist to-day of teeth filled thirty years ago even with this dirty mixture; and moreover, that the amalgams of the present day and the present mode of manipulation bear no resemblance to the material and maniplation of 1845. That used as intelligent dentists use them, they will preserve teeth that anti-amalgamites admit they would extract, and that in the hands of skilful men who know how to use, not to abuse them, they are at present invaluable.

There is a cowardly sensitiveness with some leading men in our profession about openly discussing this question, lest they be ranked with those who use amalgam almost exclusively. If there are any doubts about a point of practice, it is discreditable to a man to enforce that practice at all; but those who use amalgam discriminately ought to be as able to give as good reasons for their practice with it, as with gold, and then unite in the condemnation of the men who abuse it, an abuse which is almost as prevalent to-day as ever, even though the material is purer.

The charges made against it so long ago have only been repeated by a very few, and they mostly men whose words are more consistent than their work, while on the other hand *its abuse* is strongly condemned by those who know how to use it. If there had been any sound grounds for the charges made a quarter of a century ago, they would have gathered force, as they grew in age, with the growth of the profession. Have they done so? Quite the reverse. And is it at all likely that in the remarkable progress which has characterized the dental profession during the last twenty years, that on this one question our leading operators and thinkers in Europe and America have been blind? Far more probable that those who oppose amalgam *in toto* with the persistent virulence and prejudice of the old time, have not been able to catch up to the rapid progress around them, but are to-day worse operators and poorer thinkers than in days gone by. If we were to judge from what we see and know of such men to-day, what kind of opinion of them could we consistently form? Take gold fillings and mechanical dentistry, and we daily meet with gross abuses of both in the mouths of patients who have been for years not alone under the care of quacks and superficially educated practitioners, but under the care of men who step down from their serene altitude to expose the abuses af dentistry, and the history of whose professional lives is one of slander and conceit.

The abuse of amalgam cannot be denied; but what is there in dentistry not abused? Is there any greater imposition and sham than in the abuse of gold? Considering the immense amount of amalgam used from year to year—and what a wonder we've never heard of an amalgam epidemic mortality, though it has been used so long, so much, and so ill. I think we ought to have more independent discussion on the subject, not to ventilate the egotism or boasting of men who condemn it in print—for capital—but who use it on the sly, and make their patients believe it to be something else, but to help to free us of some of the shackles on free thought and investigation which were imposed over a quarter of a century ago, and to which some of the old practitioners are still slaves.

I here present a collection of extracted teeth badly filled with amalgam, with the history of which I am acquainted, though not one of them was filled by me; and here let me digress to say, that it is an advantageous and profitable study to investigate the history of failures. No. 1 is a specimen of a very frequent result of filling such teeth, when so badly done. In the first place, the pulp had died from congestion, and the cavity had remained unfilled for two months after it had ceased to pain, and the face had been swollen a month before. The dead pulp was not removed previous to filling nor, as you can see, was the decay half excavated, or proper preparation given to the shape and borders of the cavity. The very first principles have been ignored; a careless investigation failed to discover the full extent of the cavity, and I could judge a lap of gum had been growing *over* the edge *and into* of the bottom of the cavity. In addition to this, a cusp of an upper molar antagonized too soon, and evidently prevented the rest of the teeth meeting. Now here we have a condition of things which could not be improved upon to produce, as it did, periosteal irritation, inflammation and suppuration; a condition of things, too, which would as readily have been induced by gold or any other filling. To begin, this tooth clearly should never have been filled at all under the circumstances existing at the time; and if an attempt was made to save it, demanded considerable more care. Nos. 2 and 3 were filled in 1849, and were removed last year, being the only two in the mouth which had withstood the destructive seige of decay. These two cannot be said to be badly filled considering the lease of life they have had, but there are apparent extensions of decay, which could have been prevented by a more thorough trimming of the borders. Among the points which induce failure in the use of amalgam we may enumerate as most important: 1st, previous condition of the tooth and periosteum which would diagnose the *laisser-fair* policy, if not extraction; 2nd, imperfect preparation, consisting of thin overhanging walls of enamel under which the filling is packed, but which ultimately crack and break down from mastication, exposing an unpolished edge of enamel and an easy entrance for decay; 3rd, leaving deep fissures already decayed or discoloured, which have their issue from or into the edge of the cavity to be filled; 4th, cutting the walls at the orifice so as to run two sides to a point in a sharp angle —these often running in contiguity with depressions certain to decay, and fissures already indicating its advances; 5th, leaving the extreme outside or inside edge of the cavity sharp and unpolished; 6th, leaving the filling too high, so that when it hardens one or more cusps of antagonizing teeth bear upon it too soon; 7th, not finishing the amalgam flush with the walls in approximal cavities, leaving overhanging ledges which press upon the gums, retain particles of food in contact with it and an adjoining tooth; and in crown, bucal and labial cavities, letting the amalgam lap over the edge of the cavity, and 8th, neglecting to finish or polish the filling a few days after inserted. I here present rather a disgraceful abuse of amalgam. A first and second molar having large approximal cavities, facing each other, were filled, as you see, with a very impure amalgam, and instead of being filled separately and independently seem to have been purposely united, so that in an effort to remove one, both were removed, and cannot now be separated without filing or breaking.

I do not know any better way to demonstrate the abuses of amalgam than by the cases presented, or a better means of pointing a moral and teaching a lesson on the care demanded, even in the use of the easiest manipulating filling we possess. It is noteworthy that teeth badly filled with amalgam last longer in the aggregate—the filling often clinging to the tooth when it has only one retaining point—than those badly filled with gold; and my honest conviction is, that many calling themselves dentists would do less harm to humanity, and bring less discredit upon dentistry, by using such a filling as amalgam—which, in their hands, may save the teeth—than an article which they know they cannot use properly.

It is well here, before leaving this subject, to say that there is no excuse whatever for using amalgam in the front teeth, and that wherever it is inexpedient to insert gold, we have a temporary substitute in osteo plastic or Hill's Stopping. Approximal cavities are more liable to discolor than those situated on the crowns, and the discoloration which would be of comparatively little consequence, should it ensue, in back teeth, is an objection in conspicuous cavities.

Those who oppose amalgam *in toto* are forced to admit that they have no permanent substitute, where a soft filling is demanded, by reason of the looseness of the tooth and other circumstances; and have to admit that they extract teeth they cannot fill with gold, or temporize with other soft fillings which are only temporary. This brings us to the abuse of other plastic fillings; and here I present specimens of teeth, some of which could not be well filled with gold at the time, but others which could—which were plastered up every six months, until the edges chipping away and breaking down left the teeth only fit for the forceps. I have frequently met with vigorous healthy patients who had had amalgam fillings in the teeth for five, ten and fifteen years, who were persuaded to have them cut out as endangering the system and the teeth. The expert operator, after charging a heavy fee for cutting out the filling, exposed the pulp in several cases, inserted gold or tin foil, which in every case fell out—as boasting and conceit are not the essentials to make a gold filling hold in—and finally patched them up with osteo-plastic or Hill's Stopping. The consequence was that the teeth which, if left alone, would in all probability have lasted a lifetime, are now lying before you crownless stumps. The amount of suffering endured by the patient, the waste of time and exorbitant charge for such, would lead us to trust that a day may come when such outrageous imposition—for it is nothing less—can be made actionable, and such malpractice in the mouth be as criminal as malpractice in any other part of the body.

The abuse of oxy-chloride of zinc, Hill's Stopping, and other such plastic fillings is wide-spread, owing partly to the ignorance and quackery prevailing in a large part of the profession, and the inappreciative task of a large part of the public who neglect their teeth, and will not encourage by reasonable recompense, the efforts to do the best that can be done with gold. The human teeth were not created for dental experiment, and there has been enough of this to satisfy any one of the temporary nature of these plastic fillings. There are certain favorable constitutional conditions and circumstances in which they will last several years, but they ought never to be inserted as permanent, and teeth filled with them ought not to be considered as "finished" until replaced with a permanent material. We see a great many valuable teeth destroyed, particularly the bicuspids, from this abuse or misuse of these fillings. Patients cannot and will not run to the dentist every month in the year to have fillings replaced. A summer at the sea-side may ensure the destruction of half a dozen bone filled teeth in the same mouth. If you are not able to fill a tooth with gold, it is better—but who would do it ?—to send your patient to one who is, rather than see the teeth ruined. Better fill teeth not conspicuous, with amalgam, that will preserve them a lifetime, than with gold, which in your hands is only a deception, or with soft fillings which need renewal every few months. Plastic fillings, properly used, have been an immense blessing to the profession and the public; abused they have been a curse. In the hands of dentists who are ignorant and genuine quacks, nothing in dentistry is safe or reliable.

The discovery of a soft filling of a suitable color, as durable as gold or amalgam, and as easily introduced as the latter, would, perhaps, be a mingled blessing and a curse. To those who would use it honestly it would be a great and lasting good; but it seems to me it would bring about a degeneracy of the whole art of filling teeth, and quackery would flourish wider than ever. It would be just what the quack dentist would desire, because then he would boast that he could insert it as easily as the best operator in the land. This, however, is questionable, but the abuse of the present soft fillings in use, as previously referred to in detail, would seem to predicate no better success with a better material, should one be found.

A Surgeon General in H. M. Navy, recently showed me a white fillng in a lower molar which was inserted nearly twenty years ago by a native Chinese dentist in Hong-Kong. It had every appearance to the eye of being oxy-chloride of zinc, but it was impossible, as it had withstood mastication and the action of the fluids of the mouth so long. He allowed me to try the drill on it; it was as hard as amalgam. We know that the Chinese were famed for their cement for household purposes, and I am inclined to believe that this filling referred to above is something we do not possess. I offered to replace it with gold, *gratis*, if he would let me remove it, but he refused. It would certainly be a kind of sarcasm on the dental profession of Europe and America if we were to be indebted for the plastic filling to the investigation of Chinese Dentists. Can the American Dentists in Hong Kong give us any information on this subject? I doubt if we will find the *desideratum* in the metals. Many experiments have been tried; one Dental chemist having seven different plastic fillings in the teeth. All the oxy chlorides dissolve and decompose by mineral acids and caustic alkalies. Some which are perfectly insoluble in water are invariably dissolved in the mouth. The crystalline silicates incorporated with anhydrous zinc oxide in osteo-plastics are coarser in some than others. Guillois cement for instance, has less glass than Smith's oxy-chloride of zinc, and the glass it contains is coarser. Compounds of zinc seem to be a failure for the object desired. Some have suggested Zn. Cl. 2,6 ZnO., as the most durable oxy-chloride, if they could be properly formed. Mr. Tomes suggested oxy-chloride of magnesium some years ago, but its color, its slow setting, and its solubility in acids, make it objectionable. Peroxide of manganese with soluble glass have been tried; it is very hard and resists the action of water. Pulverized silica and gum copal, and various other preparations have been proposed, but so far all have serious objections. No suitable metal has been left untried.—*Canada Journal of Dental Science.*

